# Enhancing the Efficiency of Perovskite Solar Cells through Interface Engineering with MoS_2_ Quantum Dots

**DOI:** 10.3390/nano12173079

**Published:** 2022-09-05

**Authors:** Zhao Luo, Tan Guo, Chen Wang, Jifan Zou, Jianxun Wang, Wei Dong, Jing Li, Wei Zhang, Xiaoyu Zhang, Weitao Zheng

**Affiliations:** 1 Key Laboratory of Automobile Materials MOE, School of Materials Science & Engineering, Jilin Provincial International Cooperation Key Laboratory of High-Efficiency Clean Energy Materials, Electron Microscopy Center, Jilin University, Changchun 130012, China; 2 College of Electronic Science and Engineering, Jilin University, Changchun 130012, China

**Keywords:** perovskite, solar cell, MoS_2_, quantum dots, interface

## Abstract

The interface of perovskite solar cells (PSCs) determines their power conversion efficiency (PCE). Here, the buried bottom surface of a perovskite film is efficiently passivated by using MoS_2_ quantum dots. The perovskite films prepared on top of MoS_2_-assisted substrates show enhanced crystallinity, as evidenced by improved photoluminescence and a prolonged emission lifetime. MoS_2_ quantum dots with a large bandgap of 2.68 eV not only facilitate hole collection but also prevent the photogenerated electrons from flowing to the hole transport layer. Overall promotion leads to decreased trap density and an enhanced built-in electric field, thus increasing the device PCE from 17.87% to 19.95%.

## 1. Introduction

Perovskite solar cells (PSCs) have been widely explored in recent years due to their low cost and high efficiency [1]. Notably, inverted-structure devices have shown negligible hysteresis and high stability due to the employment of undoped hole transport materials [2]. However, their power conversion efficiency (PCE) still lags that of normal structure devices, mainly due to the energy and carrier losses at the interface between the hole transport layer (HTL) and the perovskite active layer [3]. Although replacing the typical poly(3,4-ethylenedioxythiophene)s (PEDOT:PSS) with poly[bis (4-phenyl) (2,4,6- trimethylphenyl)amine] (PTAA) can significantly suppress interface losses [4], there is still a large number of voids and defects at the buried bottom surface of the perovskite layer. This is caused by the hydrophobicity of PTAA film, which serves as a substrate affecting the growth of perovskites. Degradation is more likely to happen at trap centers than inside the crystal under illumination, an electric field, and humidity conditions. Thus, buried interfacial voids and defects will serve as decomposition centers that accelerate device efficiency decline [5]. 

The interface between the HTL and the perovskite layer determines the hole transfer rate and hole collection efficiency [6], which can be significantly improved by enhancing the coupling between layers [7]. Moreover, interface engineering with various materials, including polymers [8,9,10], large organic cations [11,12], fullerene derivatives [13,14], carbon dots [15,16], and quantum dots (QDs) [17], can also block the undesired charge carrier flow, passivate surface traps, and improve the interface contact.

Molybdenum disulfide (MoS_2_), with high charge carrier mobilities and a tunable energy band structure, is a promising low dimensional material used in optoelectronic applications. The lattice of MoS_2_ and MAPbI_3_ (MA, methylamine) perovskites match well with each other, so they can form high-quality heterojunctions via epitaxy growth [18], being beneficial for reducing non-radiative recombination. MoS_2_ has been used mainly as a hole-transporting layer or dopant in these layers [19,20]. However, it is difficult to adjust the chemical properties of most MoS_2_ used in photoelectric devices [21]. Meanwhile, stacking 2D MoS_2_ reduces interface contact and easily causes a void at the interface.

Rich chemistry and size effects make QDs unique 0D materials with huge possibilities to tune their electronic properties and enhance device interface contact. Leyla et al. utilized MoS_2_ QDs with reduce graphene oxide (RGO) hybrids as hole transport layers (HTLs) and active buffer layers. The final PCE of the normal structure PSCs with MoS_2_ quantum dot/graphene hybrids reached 20.12% [22]. Here, we engineer the interface between perovskite and PTAA using MoS_2_ QDs, which passivates the buried defects, facilitates interface contact, promotes the perovskite crystal quality, prevents reverse carrier flow, and enhances hole collection. The MoS_2_ QDs are capped with trioctyl-phosphine oxide (TOPO). With optimized MoS_2_ QD layers, the PCE of perovskite solar cells increases from 17.87% to 19.95%, demonstrating that MoS_2_ QDs are indeed promising materials for passivating the buried defects of solution-processed perovskite optoelectronic devices.

## 2. Materials and Methods

Lead iodide (PbI_2_), lead bromide (PbBr_2_), formamidinium iodide (FAI), methylamium bromide (MABr), cesium iodide (CsI), and poly[bis(4-phenyl)(2,4,6-triMethylphenyl)aMine] (PTAA) were purchased from Xi’an Polymer Light Technology Corp (China). Bathocuproine (BCP) and (6,6)-phenyl C61-butyric acid methyl ester (PCBM) were purchased from Luminescence Technology Corp (Taiwan, China). N, N-dimethylformamide (DMF), dimethyl sulfoxide (DMSO), tri-n-octylphosphine (TOP), N-methylpyrrolidone (NMP) and sulfur were purchased from Sigma-Aldrich (Sigma-Aldrich, St. Louis, MO, USA). Ammonium molybdate tetrahydrate and cysteine were purchased from Sinopharm Chemical Reagent limited corporation (Beijing, China). Chlorobenzene (CB) was purchased from J&K Scientific Corp (Beijing, China). Silver was purchased from commercial sources with high purity.

The MoS_2_ QDs were synthesized using the hydrothermal method. Ammonium molybdate tetrahydrate (80.4 mg) and cysteine (20 mg) were dissolved in 10 mL NMP, heated, and stirred at 70 °C for 20 min. Sulfur (19.0 mg) was added into 2.5 mL TOP in a glove box before being stirred and heated at 70 °C until it was fully dissolved. Then, a 333 μL sulfur solution was added to the ammonium molybdate tetrahydrate NMP solution. Stirring and heating continued through the addition of 30 μL HCl (0.1 M) until the solution was transparent. The mixture was transferred to a Teflon reactor and heated continuously at 240 °C for 24 h. After cooling down to room temperature in the reactor, the obtained solution was poured into centrifuge tubes for centrifugation. The first-time centrifugation precipitate containing sulfur and bulk MoS_2_ was abandoned. The solution was then mixed with ethanol and centrifuged at 12,000 rpm for 20 min. The target product was obtained and dispersed in DMF for device fabrication.

Perovskite precursor was prepared by dissolving 506.9 mg PbI_2_, 80.7 mg PbBr_2_, 172.2 mg FAI, and 22.4 mg MABr in 1 mL of 4:1, *v*/*v* proportion dimethylformamide (DMF) and dimethyl sulfoxide (DMSO) mixture. The solution was heated at 70 °C until it became pellucid. Next, 42.4 μL as-prepared CsI (1.5M) DMSO solution with a concentration of 1.5 M was added to the as-mixed solution, which was left on a hotplate at 70 °C for 30 min. At this point, the precursor solution was ready for perovskite film preparation.

ITO substrates were cleaned using isopropyl alcohol, acetone, chloroform, acetone, and isopropyl alcohol in sequence, and all were treated with ultrasound for 10 min. Then, the ITO substrates were blow-dried with a dry nitrogen flow and treated with a vacuum plasma cleaner for 15 min. The PTAA (1.5 mg mL^−1^, in toluene) solution was spin-coated onto the pre-cleaned ITO substrates at 4000 rpm for 30s and heated at 100 °C for 10 min. After cooling down, 30 μL MoS_2_ QDs in different concentrations were spin-coated on PTAA layers at 4000 rpm for 8 s. Immediately, 70 μL perovskite precursor solution was dropped on the prewetted substrates. A two-stage spin-coating procedure was used for perovskite coating, 1000 rpm for 10 s and 6000 rpm for 20 s. During the second spin-coating step at 6000 rpm, 120 μL chlorobenzene was quickly dropped to the center of the wet film. After annealing at 100 °C for 10 min and cooling down to room temperature, PCBM (20 mg/mL) solution was dropped on the perovskite and spin-coated at 1500 rpm for 30 s and annealed at 100 °C for 10 min. Then, a 40 μL BCP (0.5 mg/mL in isopropanol) solution was dynamically dropped on the surface of the PCBM layer at a speed of 4000 rpm for 40 s. Finally, a Ag electrode was fabricated on the BCP layer using thermal evaporation depositing. The active area could be confirmed by the ITO and Ag electrode overlapping region of 0.047 cm^−2^.

X-ray diffraction (XRD) patterns were measured with a D8 Advance-Bruker AXS X system (Bruker AXS, Karlsruhe, Germany). Transmission electron microscopy (TEM) images were taken with a JEM 2100F TEM (Japan Electronics Co., Ltd, Japan). X-ray photoelectron spectroscopy (XPS) spectra were characterized with ESCALAB 250 (Thermo Fisher Scientific, Waltham, MA, USA). A previously reported method was referred to for the characterization of the interface [6]. Scanning electron microscope (SEM) images were taken using a Hitachi SU8000 SEM (Hitachi Limited, Tokyo, Japan) at 5 kV accelerating voltage. Steady-state photoluminescence spectra (PL) were acquired using an Ocean Insight USB4000 (Ocean Optics, Dunedin, FL, USA) with a 365 nm laser as the excitation light source. Absorption spectra were measured with a Shimadzu UV-1700 spectrometer (Shimadzu corporation, Kyoto, Japan). Fourier-transform infrared spectroscopy (FTIR) was conducted using a Nicolet IS50 FT-IR (Thermo Fisher Scientific, Waltham, MA, USA). Current density–voltage (J-V) and space-charge-limited current (SCLC) curves were measured using a Keithley 2400 Source Meter (Tektronix, Beaverton, OR, USA). All the current density-voltage measurements were conducted using a solar simulator (Class AAA solar simulator, Newport Technologies, Inc., Irvine, CA, USA) External quantum efficiency (EQE) curves were measured using a Crowntech QTest Station 1000 AD (Crowntech, INC., Indianapolis, IN, USA). Capacitance–voltage (C-V) curves were tested at a constant frequency of 100 kHz. Electrochemical impedance spectroscopy (EIS) was measured using an Admiral electrochemical workstation (Admiral Instruments, Tempe, AZ, USA) from 10 Hz to 1000 kHz frequency with 0.89 V bias voltage in dark conditions.

## 3. Results and Discussion

### 3.1. Characterization of MoS_2_ QDs

The MoS_2_ QDs were prepared hydrothermally, wherein the deionized water was replaced by NMP. Due to the strong stripping ability of NMP, the formation of bulk MoS_2_ can be suppressed during the synthesis [23]. Tri-n-octylphosphine (TOP) was oxidized to Tri-n-octylphosphine oxide, capping the MoS_2_ QDs and further restricting the size of the quantum dots.

**Figure 1:**
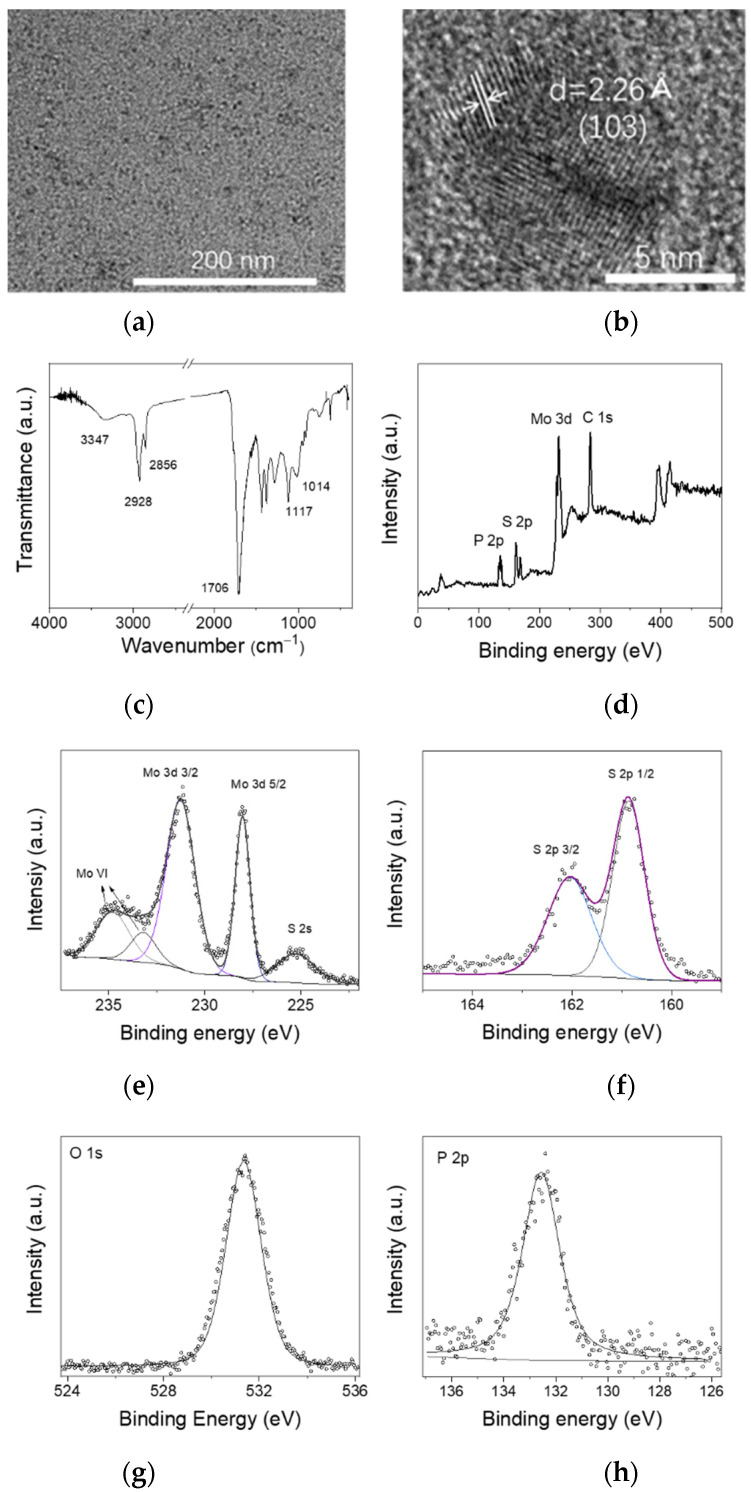
(**a**) TEM image, (**b**) high-resolution TEM images, (**c**) FTIR spectrum of the MoS_2_ QDs. (**d**) XPS survey spectrum of the MoS_2_ QDs. (**e**) High-resolution peak-fitting XPS spectrum of Mo 3*d*. (**f**) High-resolution peak-fitting XPS spectrum of S 2*p*. (**g**) High-resolution peak-fitting XPS spectrum of O 1*s*. (**h**) High-resolution peak-fitting XPS spectrum of P 2*p*.

A transmission electron microscopy (TEM) image is given as Figure 1a, showing the morphology of MoS_2_ QDs. The uniformly dispersed MoS_2_ QDs indicates their excellent polydispersity in DMF. The high-resolution TEM image (Figure 1b) shows the interplanar spacing of 2.26 Å of a near-spherical particle, which is assigned to the (103) plane of MoS_2_. The X-ray photoelectron spectroscopy (XPS) spectra were characterized to determine the chemical states and the element composition of the as-prepared MoS_2_ QDs. The peaks of Mo, S, P, and C are clearly observable in the survey spectrum (Figure 1c). Figure 1d shows characteristic peaks at 162.05 eV and 160.9 eV, which are relevant to S 2*p* 1/2 and S 2*p* 3/2 orbitals, respectively. Two strong peaks located at 228.05 eV and 231.35 eV in Figure 1e are assigned to Mo 3*d* 5/2 and Mo 3*d* 3/2 orbitals, respectively, proving the existence of Mo^4+^. There are two peaks at 234.8 and 235.95 eV assigned to Mo^6+^ 3*d*5/2 and Mo^6+^ 3*d*3/2. The higher valance resulted from the partial oxidation of MoS_2_ QD with the remaining oxygen in the Teflon-lined stainless-steel autoclave during the reaction [24,25]. Moreover, the atomic ratio of S to Mo is calculated as ~1.89. In addition, the P element from TOP can also be detected from the XPS, as shown in Figure 1g,h. The P 2*p* and the O 1*s* signal prove the formation of the oxidation product of TOP. As shown in the Fourier transform infrared (FTIR) spectrum of MoS_2_ QDs (Figure 1f), the four dominating peaks at 3347, 2928, 2856, and 1706 cm^−1^ can be observed, corresponding to NH_2_, CH_2_, CH_3_, and C=O groups, respectively. The peaks at 1117 and 1014 cm^−1^ are assigned to P=O [26,27], indicating that the oxidation product of TOP serves as the surface ligand of MoS_2_ QDs, which is in line with the XPS results.

**Figure 2:**
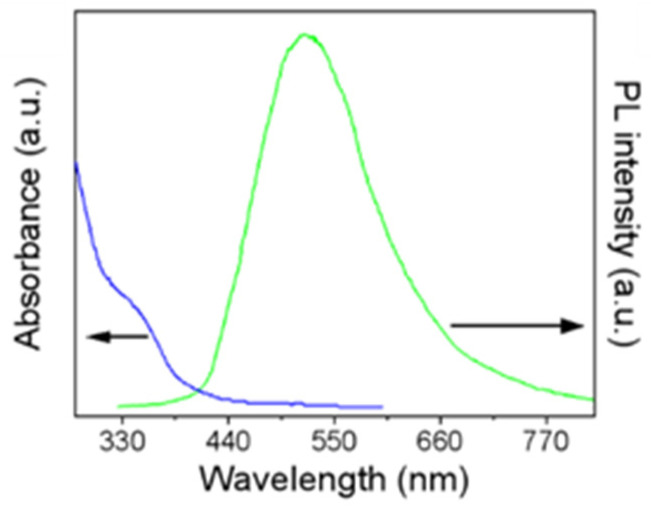
Absorption and PL spectra of MoS_2_ QD colloid.

Figure 2 shows the UV-visible absorption and photoluminescence (PL) spectra of MoS_2_ QDs. The PL peak and absorption peak are located at 518 nm and 343 nm, respectively. The band gap of MoS_2_ QDs can be calculated from the absorption spectrum according to Lambert–Beer’s law, which is described as:

(*αhν*)^2^ = *BK*^2^ (*hν*−*E_g_*) (1)

where *B* and *K* are proportionality constants, *α* is the absorption coefficient, *E_g_* is the band gap energy, and *hν* is the energy of incident photons. Based on the quantum confinement effect [28], the *E_g_* of the as-prepared MoS_2_ QDs is about 2.68 eV.

### 3.2. Characterization of Perovskite Films

**Figure 3:**
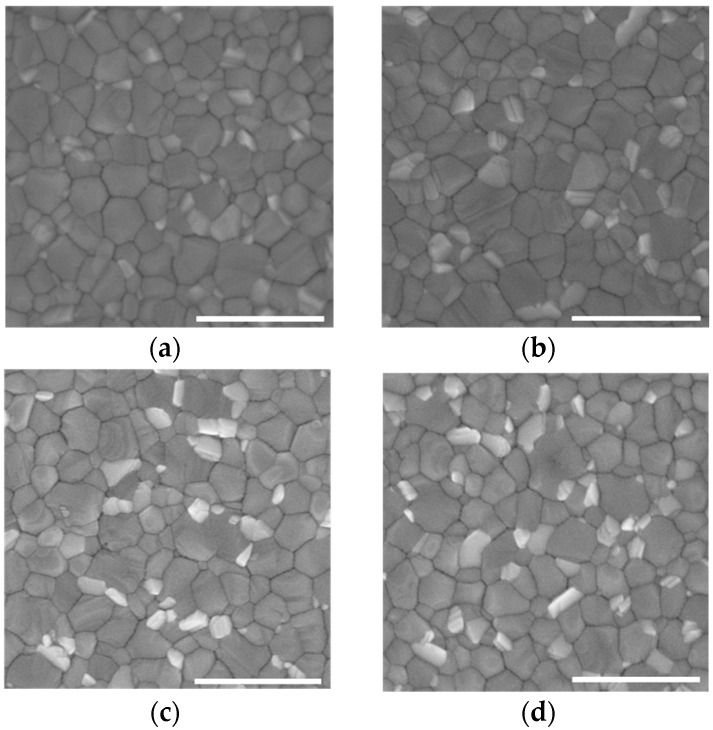
SEM images of perovskite films. (**a**) The control perovskite film. (**b**) Perovskite film treated with 0.075 mg/mL MoS_2_ QDs. (**c**) Perovskite film treated with 0.10 mg/mL MoS_2_ QDs. (**d**) Perovskite film treated with 0.15 mg/mL MoS_2_ QDs. Scale bar: 1 μm.

**Figure 4:**
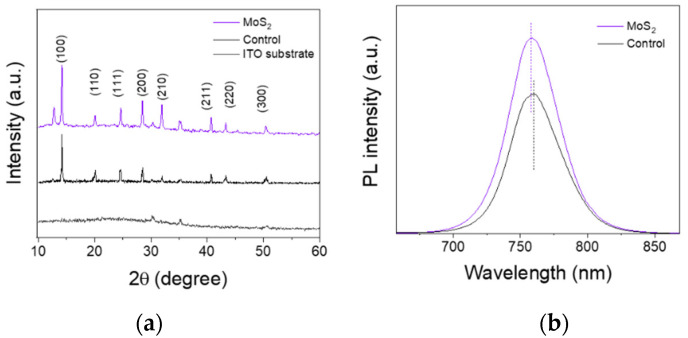

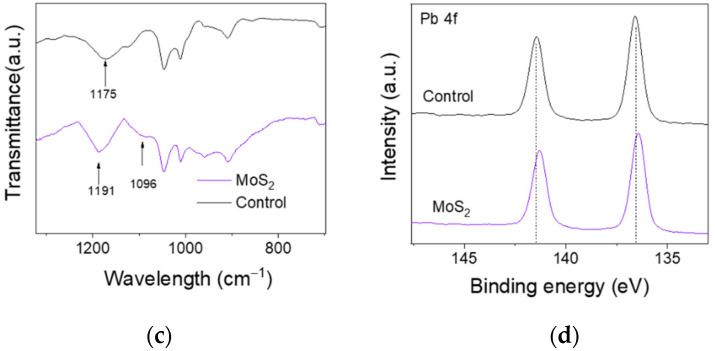
(**a**) XRD patterns, (**b**) PL spectra, and (**c**) FTIR spectra of perovskite film with and without MoS_2_. (**d**) High-resolution Pb 4*f* peaks of the perovskite films with and without MoS_2_ QDs.

To determine the influence of MoS_2_ QDs on perovskite films, the top-view SEM images shown in Figure 3 were compared. It was noted that the film with MoS_2_ QD modification exhibits more homogeneous grains, and some light-colored particles are distributed on top of the films, which can be observed as PbI_2_ by X-ray diffraction (XRD) patterns in Figure 4a [6].

The XRD patterns of the perovskite films deposited on the PTAA and PTAA/MoS_2_ QD substrate are shown in Figure 4a. The perovskite films both present characteristic diffraction peaks [29]. There are no perovskite peak position shifts, indicating that the Mo or S atom did not diffuse into the perovskite crystal and enable doping. Compared with the control film, the perovskite film deposited on the PTAA/MoS_2_ QD substrate suggests a peak at 12.5° belonging to (001) crystal planes of PbI_2_, which is consistent with the SEM analysis [30,31,32]. Moreover, the increased intensity of the peaks corresponding to perovskites evidently indicates a higher quality of perovskite films and crystal-oriented growth. The promoted peak intensity can be attributed to the interaction between MoS_2_ QDs and perovskites. MoS_2_ QDs served as a growth template and induced crystal growth [33]. The FTIR spectra of the perovskite film with MoS_2_ QDs and without are shown in Figure 4c. The peak shifts from 1175 to 1191 cm^−1^, which overlaps P=O peaks. Moreover, there is a new peak at 1096 cm^−1^ belonging to P=O. Compared to the QD FTIR spectrum, the peak shifts from 1014 cm^−1^ to 1096 cm^−1^.

To determine the chemical effects of MoS_2_ QD modification, the buried interfaces were characterized using XPS [6]. The XPS results of the MoS_2_-QD-modified perovskite and control film are shown in Figure 4d. According to the XPS spectra of the P*b 4f r*egion, the binding energy decreased from 136.58 eV and 141.45 eV to 136.40 eV and 141.28 eV, respectively. The lower binding energy of Pb could be ascribed to the strong interaction between the QD modification and Pb through the Lewis acid–base interaction. The XPS results confirm the interaction between the MoS_2_ QD layer and perovskite films, indicating increased electron density [34]. The XPS results confirm the interaction between the MoS_2_ QD layer and perovskite films.

To further characterize the effects of MoS_2_ QDs on the photophysical properties of perovskites, we observed the steady-state PL spectra of the perovskite films with and without MoS_2_ QDs, as shown in Figure 4b. It is noted that the PL peak blue-shifts from 761 nm to 759 nm with the assistance of MoS_2_ QDs, indicating a lower density of trap states [35,36]. Meanwhile, the perovskite film with MoS_2_ QDs presents a much higher PL intensity, which means less nonradiative recombination losses [37]. Combined with the XPS results, the Pb 4*f* peaks shift toward the lower binding energy, demonstrating that the suppressed nonradiative recombination in the perovskite films is related to the interaction between the QDs and perovskites, which is responsible for the reduced traps and improved PL.

**Figure 5:**
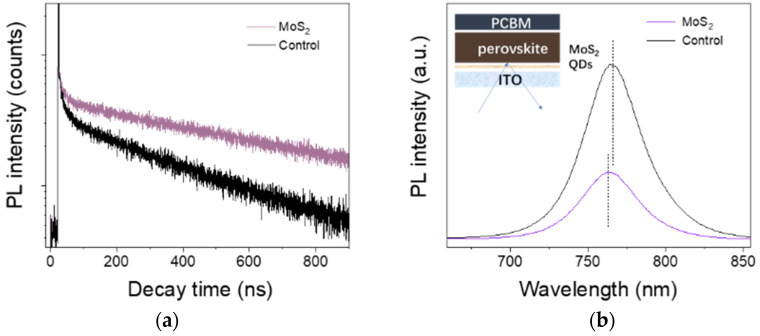
(**a**) TRPL curves of perovskite films deposited on the substrates with and without MoS_2_ QDs. (**b**) The PL spectra of perovskite films with and without MoS_2_ QDs, where the schematic diagram of the test detail is given as an inset.

To evaluate the effects of MoS_2_ QDs on the carrier dynamics, time-resolved photoluminescence (TRPL) measurement was employed. As shown in Figure 5a, TRPL curves were fitted using a bi-exponential function described as:

*I(t)* = *I_0_* + *A*_1_
*exp*(−*t/τ*_1_) + *A*_2_
*exp*(−*t/τ*_2_) (2)

where *τ*_1_ and *τ*_2_ represent the fast decay time constant and the slow time constant, respectively, and *A*_1_ and *A*_2_ are the amplitudes of the fast and slow decay processes [38,39]. The average carrier lifetime (*τ*_ave_) was acquired through equation *τ*_ave_ = *τ*_1_*·f*_1_+ *τ*_1_*·f*_2_. 

The fitting relevant parameters are summarized in Table 1. The proportion of the fast decay component *τ_1_* representing the surface trap-assisted nonradiative recombination decreased from 52% to 40.5%. Moreover, the prolonged average carrier lifetime (398.8 ns) compared with that of the control film (175.3 ns) further demonstrates the promoted photophysical properties of suppressing the nonradiative recombination processes.

We also noted the formation of PbI_2_ on the surface and at the grain boundaries. The PL peaks were compared to further understand the influence of PbI_2_ in Figure 5b. When the PCBM film as electron transport layer (ETL) was deposited on top of the perovskite films, the sample with QDs showed significantly weaker PL, demonstrating that carriers were extracted more efficiently the pure perovskite film. It is reported that the PbI_2_ located at the surface of the perovskite film could heal the halide vacancy forming type-I band alignment and reduce the trap state density [36,40,41,42,43].

### 3.3. Perovskite Solar Cell Performances

**Figure 6:**
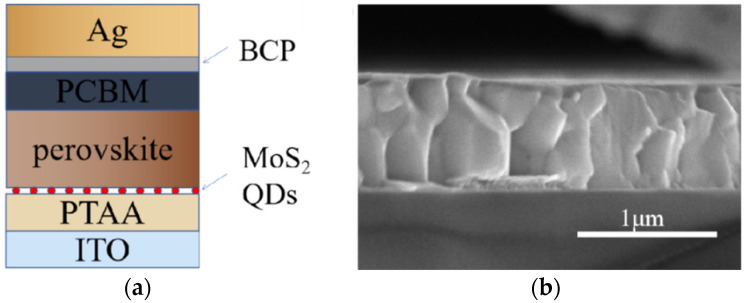
(**a**) A schematic diagram of the PSCs structure. (**b**) Cross-sectional SEM image of the control device.

Typical perovskite solar cells (PSCs) were fabricated with the structure of ITO/PTAA/perovskite/PCBM/BCP/Ag, as shown in Figure 6a. A cross-section SEM of the device is shown in Figure 6b.

**Figure 7:**
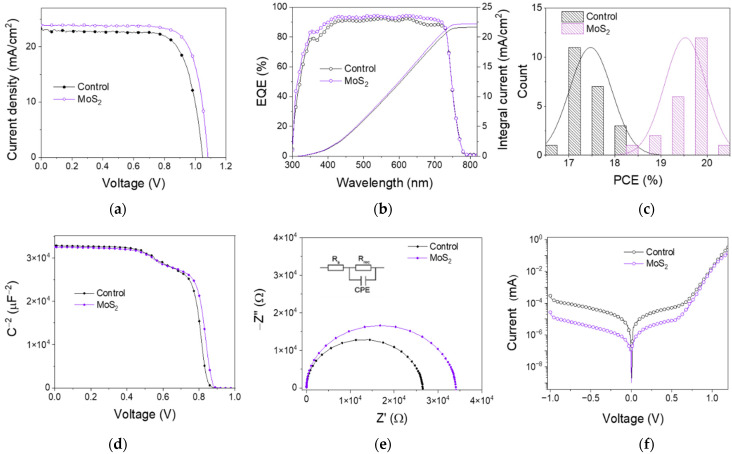
(**a**) *J–V* curves of control device and devices treated with MoS_2_ QDs at different concentrations. (**b**) IPCE measurement of control device and device treated with MoS_2_ QDs. (**c**) Measurement for PCE of devices with and without MoS_2_, where 22 cells were collected for each batch. (**d**) Capacitance–voltage curves of control device and device treated with MoS_2_ QDs. (**e**) Nyquist plots of control device and device treated with MoS_2_ QDs. Inset: An equivalent circuit model of the devices. (**f**) Dark *J–V* curves of control device and device treated with MoS_2_ QDs.

The current density–voltage (*J–V*) curves of the devices are provided in Figure 7a, while the device parameters are summarized in Table 2. As expected, due to the QDs’ passivation effect, the V_oc_ (open circuit voltage) and *J*_sc_ (short circuit current) were enhanced from 1.05 to 1.08 V and 23.25 mA/cm^2^ to 23.79 mA/cm^2^, respectively. Consequently, a relatively superior performance with a power convention efficiency (PCE) from 17.87% to 19.95% was achieved. Figure 7c shows the statistic efficiency distribution. The current densities integrated from the incident photon to converted electron spectra (IPCE) increased from 21.67 to 22.15 mA/cm^2^ with the MoS_2_ modification, which is consistent with the increased *J*_sc_. The enhancement of IPCE (Figure 7b) in the short wavelength part could be attributed to the MoS_2_ QDs at the HTL interface acting as an interface passivator. Furthermore, the increase in long wavelength conversion may be related to the passivation effect of lead iodide at the electron collection side.

To further investigate the influence of MoS_2_ QD modification on the carrier extraction/transport in devices, dark capacitance–voltage curves were measured, as shown in Figure 7d. The Mott–Schottky relationship can be described as follows:

*1/C*^2^ = *2(V*−*V_bi_*−*kT/e)*/*(εε*_0_*eN_D_)* (3)

where *V_bi_* is the voltage of the built-in electric field, *V* is the applied voltage, *C* is the capacitance, *k* is the Boltzmann’s constant, *T* represents the temperature, and *N_D_* is the donor density. The fitting results show that the *V_bi_* of the device with MoS_2_ QDs increased from 0.84 V to 0.86 V. The enhanced driving force to separate photogenerated carriers accounts for the higher *V_oc_* [44,45].

Electrochemical impedance spectroscopy (EIS) under 0.85 V bias voltage and dark conditions was performed to further explore the charge transfer and recombination progress [1,46]. Nyquist plots of devices with and without MoS_2_ are presented in Figure 7e. An equivalent circuit model is depicted as an inset where series resistance (R_s_) and recombination resistance (R_rec_) represent the resistance corresponding to the behavior of carrier transport and recombination, respectively. The plot for devices with MoS_2_ QDs clearly shows a larger diameter of the semicircle than that of the control device, indicating a larger recombination resistance and chemical capacitance, which corroborates the suppression of carrier recombination and an improvement in transfer and extraction efficiency. This phenomenon can be ascribed to the passivation of the defects and band alignment at the interface. Dark *J*–*V* curves were measured to confirm the buffer function of MoS_2_ QD modification. As shown in Figure 7f, current leakage of the device was reduced by nearly more than one order of magnitude with the introduction of MoS_2_ QDs. The remarkable decrease in the current leakage demonstrates that MoS_2_ QDs as a buffer layer prevent the photogenerated electrons from flowing to the hole transport layer [47,48,49].

**Figure 8:**
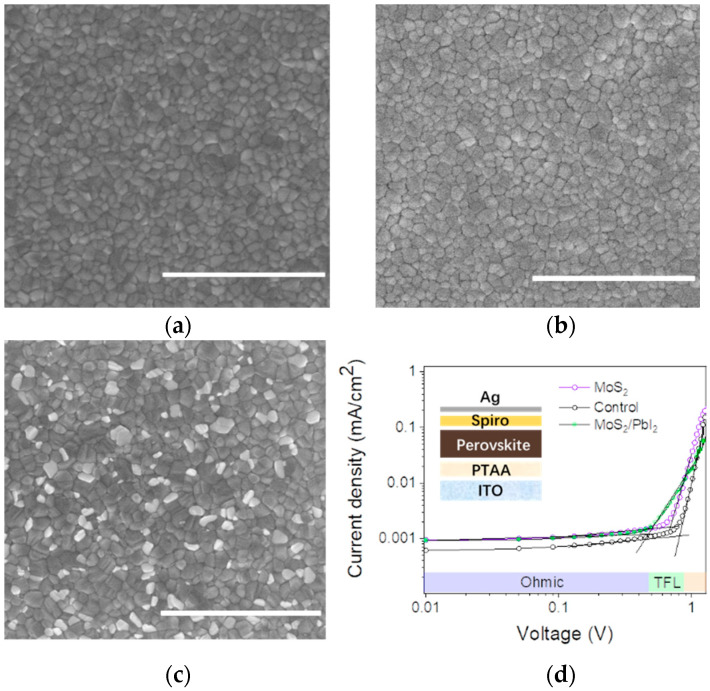
(**a**,**b**) The films after mechanical exfoliation treatment without PbI_2_ morphology. (**c**) The film with MoS_2_ and PbI_2_. Scale bar in the SEM images is 3 μm. (**d**) SCLC measurement device treated with MoS_2_, with and without PbI_2_. Inset: the hole-only devices structure diagram.

To further individually clarify the effect of PbI_2_ and MoS_2_, we adopted the mechanical exfoliation method to remove the PbI_2_ on the top surface of the perovskite films [50]. The films without PbI_2_ SEM images are shown in Figure 8a–c. Based on the treated films, we fabricated hole-only devices with and without MoS_2_.

Subsequently, space-charge-limited current (SCLC) measurements were carried out to estimate the trap densities by employing hole-only device configurations (ITO/PTAA/Perovskite/Spiro-OMeTAD/Ag). According to the SCLC mode, the defect state density is determined by the following equation [51,52]:

*n_t_* = (2*εε*_0_*V_TFL_*)/(*eL*^2^) (4)

where *n_t_* is trap density; *V_TFL_* represents trap-filled limited voltage*;* e is elementary charge; and *ε* and *ε_0_* are the permittivity of perovskite films and the vacuum permittivity, respectively. 

Figure 8d suggests that the V_TFL_ for the optimized device is 0.70 V compared to the control device’s 0.81 V. Without PbI_2_, the defect density is reduced from 9.6 × 10^15^ to 8.3 × 10^15^, which could individually prove the MoS_2_ effects. The reduced trap density demonstrates that the introduction of MoS_2_ QDs can effectively suppress carrier recombination, which is consistent with the prolonged carrier lifetime and improved device performance. Furthermore, the films with MoS_2_ and PbI_2_ show the least defective state density, which is 6.9 × 10^15^. These results prove the synergistic passivation of MoS_2_ and PbI_2_.

**Figure 9:**
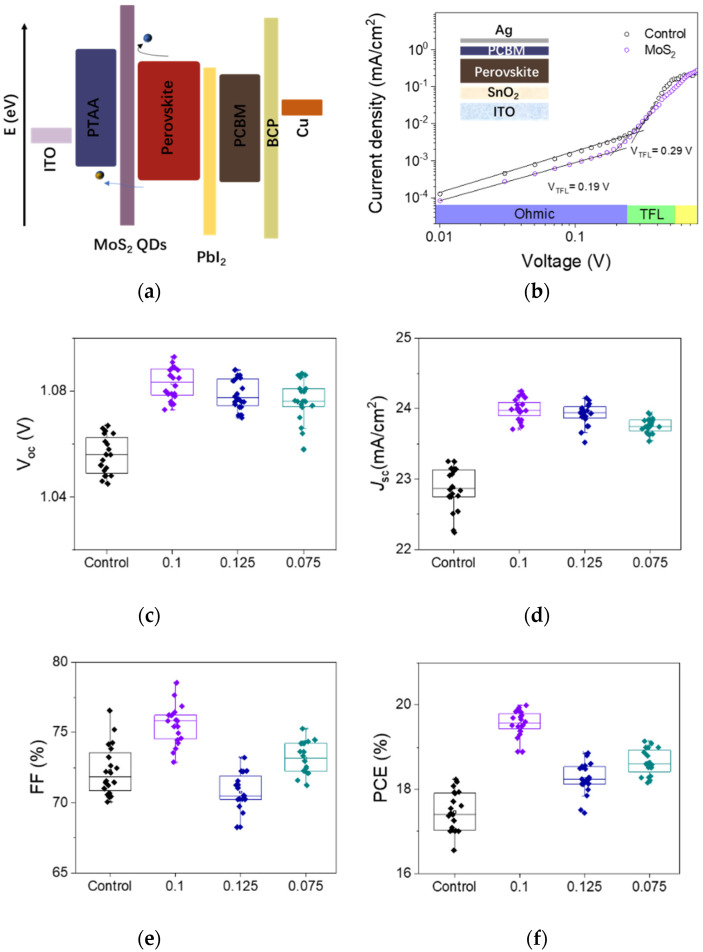
(**a**) Schematic diagram of band energy. (**b**) SCLC measurement of control device and device treated with MoS_2_. Inset: the electron-only devices structure diagram. (**c**–**f**) The statistics of *V_oc_*, *J_sc_*, FF, and PCEs of 20 devices for each fabrication condition.

Electron-only devices were fabricated, and the trap state density for electron transfer was calculated in Figure 9b. The defect state density decreased from 1.25 × 1016 cm^−3^ to 0.82 × 1016 cm^−3^. As shown in the schematic diagram of band energy (Figure 9a), the PbI2 on the electron transfer layer side could facilitate the extraction of electrons and reduce the defect state density. Thus, we assume that the MoS2 on the buried interface and the PbI2 on the top interface promote hole and electron transfer, respectively.

**Figure 10:**
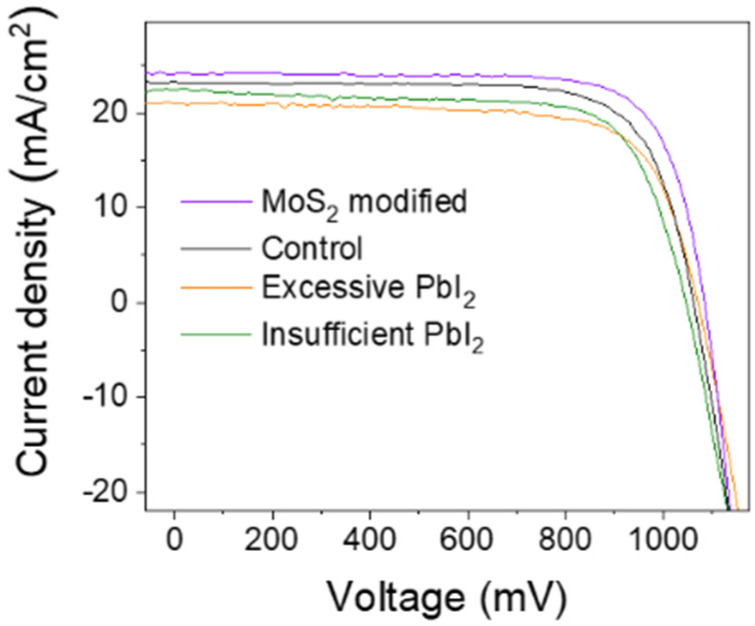
*J–V* curves of device with 5% excess PbI_2_, device with MoS_2_, control device, and MoS_2_ modified device.

We introduced 5% excess PbI2 and reduce the 5% proportion PbI2 in the precursor on the base of the component proportion in the manuscript and then respectively fabricated devices, namely excessive PbI2 and insufficient PbI2. The devices with 5% excessive PbI2, insufficient PbI2 and control device performances are compared in the Figure 10.

The device parameters are shown in Table 3. The device introduced to 5% excess PbI2 in the precursor showed significant current density decline, while the device with excessive PbI2 showed enhanced open voltage compared to the other devices. The con-trol device suggests higher current density compared to the insufficient device and ex-cess device, which indicates that a proportion of the control devices are best without modification.

Based on the above experiment and analysis, a conclusion could be drawn: the MoS_2_ QDs could passivate the defect through the interaction without PbI_2_, while the PbI_2_ induced by MoS_2_ reduced the trap density further. Simply adding excessive PbI_2_ could not achieve higher efficiency and lower trap density. The box plot statistics of Voc, J_sc_, FF, and PCEs of 20 devices for each fabrication condition are shown in Figure 9c–f. The MoS_2_-modified device without PbI_2_ showed lower defect density. In a word, the PbI_2_ induced by MoS_2_ QDs and MoS_2_ both play a role in passivation. Under synergistic effects, the current density and open voltage were both improved.

## 4. Conclusions

In summary, we employed MoS_2_ QDs as interface passivating agents to improve the performance of perovskite solar cells. It was found that MoS_2_ QDs can improve perovskite crystallinity and passivate interfacial defects, which prolongs carrier lifetimes and suppresses carrier recombination. Moreover, due to the wide bandgap of 2.6 eV, the MoS_2_ QDs served as a buffer layer, reducing the shunting paths. Benefitting from the suppressed nonradiative recombination and reduced leakage current, the device performance of PSCs improved from 17.87% to 19.95% after MoS_2_ QD modification. This work provides an exploration of QDs for PSC applications, specifically for perovskite film modification and buried interface passivation.

## Figures and Tables

**Table 1:** Relevant parameters fitted using the TRPL curves.

Samples	*τ*_1_ (ns)	*f*_1_ (%)	*τ*_2_ (ns)	*f*_2_ (%)	*τ*_ave_ (ns)
Without MoS_2_	11.7	52.6	354.6	47.4	175.3
With MoS_2_	11.5	40.5	662.5	59.5	398.8

**Table 2:** Relevant device parameters obtained from *J-V* curves.

Samples	*J*_cs_ (mA/cm^2^)	V_oc_ (V)	FF (%)	PCE (%)
0 mg/mL MoS_2_	22.89	1.05	74.35	17.87
0.075 mg/mL MoS_2_	23.30	1.06	73.81	18.23
0.100 mg/mL MoS_2_	23.79	1.08	77.64	19.95
0.125 mg/mL MoS_2_	23.99	1.07	72.07	18.50

**Table 3:** Relevant device parameters obtained from *J-V* curves.

Samples	*J*_cs_ (mA/cm^2^)	V_oc_ (V)	FF (%)	PCE (%)
0 mg/mL MoS_2_	24.07	1.085	76.39	19.95
0.075 mg/mL MoS_2_	23.25	1.063	73.64	18.20
0.100 mg/mL MoS_2_	22.35	1.049	71.01	17.29
0.125 mg/mL MoS_2_	21.42	1.072	72.51	16.32

## Data Availability

The data that support the findings of this study are available from the corresponding author upon reasonable request.
